# Quantum steerability based on joint measurability

**DOI:** 10.1038/s41598-017-15910-8

**Published:** 2017-11-17

**Authors:** Zhihua Chen, Xiangjun Ye, Shao-Ming Fei

**Affiliations:** 1 Department of Mathematics, College of Science, Zhejiang University of Technology, Hangzhou, 310023 China; 2 Key Laboratory of Quantum Information, University of Science and Technology of China, CAS, Hefei, 230026 China; 3 Synergetic Innovation Center of Quantum Information and Quantum Physics, University of Science and Technology of China, Hefei, 230026 China; 4 School of Mathematical Sciences, Capital Normal University, Beijing, 100048 China; 5 Max-Planck-Institute for Mathematics in the Sciences, 04103 Leipzig, Germany

## Abstract

Occupying a position between entanglement and Bell nonlocality, Einstein-Podolsky-Rosen (EPR) steering has attracted increasing attention in recent years. Many criteria have been proposed and experimentally implemented to characterize EPR-steering. Nevertheless, only a few results are available to quantify steerability using analytical results. In this work, we propose a method for quantifying the steerability in two-qubit quantum states in the two-setting EPR-steering scenario, using the connection between joint measurability and steerability. We derive an analytical formula for the steerability of a class of X-states. The sufficient and necessary conditions for two-setting EPR-steering are presented. Based on these results, a class of asymmetric states, namely, one-way steerable states, are obtained.

## Introduction

Quantum nonlocality, EPR-steering and quantum entanglement are important quantum correlations. EPR-steering, which was originally presented by Schrodinger in the context of the famous Einstein-Podolsky-Rosen (EPR) paradox^1^, lies between quantum nonlocality and quantum entanglement, which means that one observer, by performing a local measurement on one’s subsystem, can nonlocally steer the state of the other subsystem. Recently EPR-steering was reformulated by Wiseman *et al*. who described the hierarchy among Bell nonlocality, EPR-steering and quantum entanglement^2^. EPR-steering has been shown to be advantageous for quantum tasks such as randomness generation, subchannel discrimination, quantum information processing and one-sided device-independent processing in quantum key distributions^3–7^.

Many efforts have been made to detect and measure EPR-steering. Some steering inequalities based on uncertainty relations^8–13^, inequalities based on steering witnesses and the Clauser-Horne-Shimony-Holt (CHSH)-like inequality, and geometric Bell-like inequalities *et al*.^14–20^ are constructed to diagnose the steerability, are usually necessary conditions. In addition to inequalities, all-versus-nothing proof without inequalities, were also presented to detect steerability^21^. However only a few methods are available to quantify EPR-steering based on maximal violation of steering inequalities^22^, steering weight^23^ and steering robustness. In these cases semi-definite programming is necessary to calculate the measures. Recently, the radius of a super quantum hidden state model was proposed to evaluate the steerability^24^ by finding the optimal super local hidden states. Nevertheless, it is formidably difficult to find the optimal super quantum hidden states. A critical radius was proposed via the geometrical method, and the critical radius of T-states was calculated explicitly^25^. The closed formulas for steering were derived in two- and three-measurement scenarios^26^, which is the case in which Alice and Bob are both allowed to measure the observables at their own sites. It has been proven that one-to-one mapping exists between the joint measurability and the steerability of any assemblage^27–30^. Using the connection between steering and joint measurability, the closed formula of the measure for two-setting EPR-steering of Bell-diagonal states was given^31^. However, for any two-qubit quantum states, one still lacks a closed formula for the steerability problem, even for a 2-setting scenario.

Different from Bell nonlocality and quantum entanglement, steering exhibits asymmetric features, as proposed by Wiseman *et al*.^2^. There exist quantum states ${\rho }_{AB}$, for which Alice can steer Bob’s state but Bob can not steer Alice’s state, or vice versa. This distinguishing feature could be useful for some one-way quantum information tasks such as quantum cryptography, but until recently only a few asymmetric states have been proposed and experimentally demonstrated^24,32–34^.

In this work, we investigate the analytical formula for quantification of EPR-steering and obtain the necessary and sufficient condition of steerability for a class of quantum states. The asymmetric feature of EPR-steering is also investigated.

## Setting up the stage

Consider a bipartite qubit system ${\rho }_{AB}$ shared by Alice and Bob with reduced density states *ρ*_*A*_ and *ρ*_*B*_. Alice performs positive-operator-valued measures (POVMs) ${{\rm{\Pi }}}_{\kappa |\overrightarrow{n}}$ on subsystem *A*, where ${{\rm{\Pi }}}_{\kappa |\overrightarrow{n}}=\frac{1}{2}({{\rm{I}}}_{2}+{(-\mathrm{1)}}^{\kappa }\overrightarrow{n}\cdot \overrightarrow{\sigma }),$ I_2_ is the identity matrix and $\overrightarrow{\sigma }=({\sigma }_{x},{\sigma }_{y},{\sigma }_{z})$ are the Pauli matrices. Alice obtains the result $\kappa \mathrm{\ (}\kappa =\mathrm{0,1)}$ when measuring along the direction $\overrightarrow{n}\mathrm{.}$ Bob’s unnormalized conditional state is ${\tilde{\rho }}_{\kappa |\overrightarrow{n}}={{\rm{Tr}}}_{A}[{\rho }_{AB}({{\rm{\Pi }}}_{\kappa |\overrightarrow{n}}\otimes {{\rm{I}}}_{2})]$. Bob’s unconditional state ${\rho }_{B}={{\rm{Tr}}}_{A}{\rho }_{AB}=\sum _{\kappa }{\tilde{\rho }}_{\kappa |\overrightarrow{n}}$ remains unchanged under any measurement direction. A state assemblage ${\tilde{\rho }}_{\kappa |\overrightarrow{n}}$ is unsteerable if there exists a local hidden state model (LHSM) with the state ensemble of ${p}_{i}{\rho }_{i}$ satisfying ${\tilde{\rho }}_{\kappa |\overrightarrow{n}}=\sum _{i}P(\kappa |\overrightarrow{n},i){p}_{i}{\rho }_{i}$, where ${\rho }_{B}=\sum _{i}{p}_{i}{\rho }_{i}$ and $\sum _{\kappa }P(\kappa |\overrightarrow{n},i)=1.$ The quantum state ${\rho }_{AB}$ is unsteerable from *A* to *B* if for all local POVMs, the state assemblages are all unsteerable. The quantum state *ρ*_*AB*_ is steerable from *A* to *B* if there exist measurements in Alice’s case that produce an assemblage that demonstrates steerability.

The corresponding local hidden state model and the joint measurement observables are connected through ${O}_{\kappa |\overrightarrow{n}}=\frac{1}{\sqrt{{\rho }_{B}}}{\tilde{\rho }}_{\kappa ,\overrightarrow{n}}\frac{1}{\sqrt{{\rho }_{B}}}$ and ${G}_{i}=\frac{1}{\sqrt{{\rho }_{B}}}{p}_{i}{\rho }_{i}\frac{1}{\sqrt{{\rho }_{B}}}$ by the one-to-one mapping between the joint measurement problem and the steerability problem, whenever *ρ*_*B*_ is invertible^27^. The steerability can be detected through the joint measurability of the observables.

Two-setting steering scenario: Any two-qubit quantum state can be expressed by ${\rho }_{AB}=({{I}}_{4}+\overrightarrow{a}\cdot \overrightarrow{\sigma }\otimes$ ${{I}}_{2}+{{I}}_{2}\otimes \overrightarrow{b}\cdot \overrightarrow{\sigma }+\sum _{i}^{3}{c}_{i}{\sigma }_{i}\otimes {\sigma }_{i}\mathrm{)/4}$ under local unitary equivalence, where $\overrightarrow{a},\overrightarrow{b},\overrightarrow{c}\in {R}^{3}$, ${\sigma }_{1}={\sigma }_{x}$, ${\sigma }_{2}={\sigma }_{y}$, ${\sigma }_{3}={\sigma }_{z}$, $\overrightarrow{\sigma }=\{{\sigma }_{1},{\sigma }_{2},{\sigma }_{3}\}$, $C={\rm{Diag}}\{{c}_{1},{c}_{2},{c}_{3}\}$ is the correlation matrix.

When Alice performs two sets of POVMs ${{\rm{\Pi }}}_{\kappa |{\overrightarrow{n}}_{i}}=({{I}}_{2}+{(-\mathrm{1)}}^{\kappa }{\overrightarrow{n}}_{i}\cdot \overrightarrow{\sigma }\mathrm{)/2}$ $(i=\mathrm{0,}\,\mathrm{1,}\,\kappa =\mathrm{0,}\,\mathrm{1)}$ on *A* with ${\overrightarrow{n}}_{i}=({\rm{s}}{\rm{i}}{\rm{n}}{\alpha }_{{\rm{i}}}{\rm{c}}{\rm{o}}{\rm{s}}{\beta }_{{\rm{i}}},{\rm{s}}{\rm{i}}{\rm{n}}{\alpha }_{{\rm{i}}}{\rm{s}}{\rm{i}}{\rm{n}}{\beta }_{{\rm{i}}},{\rm{c}}{\rm{o}}{\rm{s}}{\alpha }_{{\rm{i}}}),$ Bob’s unnormalized conditional states are ${\tilde{\rho }}_{\kappa |{\overrightarrow{n}}_{i}}={\rm{Tr}}[{\tilde{\rho }}_{\kappa |{\overrightarrow{n}}_{i}}]({{\rm{I}}}_{2}+$ ${(-\mathrm{1)}}^{\kappa }{\overrightarrow{s}}_{\kappa ,i}\cdot \overrightarrow{\sigma }\mathrm{)/2}$, where ${\rm{Tr}}[{\tilde{\rho }}_{\kappa |{\overrightarrow{n}}_{i}}]=\mathrm{(1}+{(-\mathrm{1)}}^{\kappa }\overrightarrow{a}\cdot {\overrightarrow{n}}_{i}\mathrm{)/2}$ and ${\overrightarrow{s}}_{\kappa ,i}=(\overrightarrow{b}+{(-\mathrm{1)}}^{\kappa }C\cdot {\overrightarrow{n}}_{i}\mathrm{)/(2}{\rm{Tr}}[{\tilde{\rho }}_{\kappa |{\overrightarrow{n}}_{i}}])$. When $|b|\ne \mathrm{1,}$ the measurement assemblages are

$$\begin{array}{rcl}{O}_{\kappa }({x}_{i},{\overrightarrow{g}}_{i}) & = & \frac{1}{\sqrt{{\rho }_{B}}}\,{\tilde{\rho }}_{\kappa |{\overrightarrow{n}}_{i}}\,\frac{1}{\sqrt{{\rho }_{B}}}=\frac{1}{2}\mathrm{((1}+{(-\mathrm{1)}}^{\kappa }{x}_{i}){{\rm{I}}}_{2}+{(-\mathrm{1)}}^{\kappa }{\overrightarrow{g}}_{i}\cdot \overrightarrow{\sigma }),\end{array}$$

where $${\overrightarrow{g}}_{i}=U\,{\overrightarrow{n}}_{i},$$ $${x}_{i}=V\,{\overrightarrow{n}}_{i}$$ with

$$U=\frac{\overrightarrow{b}\,{\overrightarrow{a}}^{T}}{|b{|}^{2}-1}+\frac{(-1+\sqrt{1-|b{|}^{2}})\overrightarrow{b}\,{\overrightarrow{b}}^{T}C}{|b{|}^{2}(|b{|}^{2}-\mathrm{1)}}+\frac{C}{\sqrt{1-|b{|}^{2}}},$$

and $V=\frac{{\overrightarrow{a}}^{T}-{\overrightarrow{b}}^{T}C}{1-|b{|}^{2}}\mathrm{.}$ Thus, ${\{{\tilde{\rho }}_{\kappa |{\overrightarrow{n}}_{i}}\}}_{\kappa ,i}$ are unsteerable assemblages if and only if ${\{{O}_{\kappa }({x}_{i},{\overrightarrow{g}}_{i})\}}_{\kappa ,i}$ are jointly measurable^35–37^, namely,

1 $$(1-{F}_{{x}_{0}}^{2}-{F}_{{x}_{1}}^{2})(1-\frac{{x}_{0}^{2}}{{F}_{{x}_{0}}^{2}}-\frac{{x}_{1}^{2}}{{F}_{{x}_{1}}^{2}})-{({\overrightarrow{g}}_{0}\cdot {\overrightarrow{g}}_{1}-{x}_{0}{x}_{1})}^{2}\le \mathrm{0,}$$

where $${F}_{{x}_{i}}=\frac{1}{2}(\sqrt{{\mathrm{(1}+{x}_{i})}^{2}-{g}_{i}^{2}}+\sqrt{{\mathrm{(1}-{x}_{i})}^{2}-{g}_{i}^{2}}),$$ $${g}_{i}=|{\overrightarrow{g}}_{i}\mathrm{|.}$$

(1) gives rise to the condition for Alice to steer Bob’s state. If Bob performs two sets of POVMs ${{\rm{\Pi }}}_{\kappa |{\overrightarrow{n}}_{i}}$ on his system to steer Alice’s state, the corresponding condition can be similarly written by changing $\overrightarrow{a}\to \overrightarrow{b}$, $\overrightarrow{b}\to \overrightarrow{a}$ and $C\to {C}^{T}$ in (1).

However, it is generally quite difficult to address condition (1) and obtain explicit conditions to judge the steerability for an arbitrary given two-qubit state. For Bell-diagonal states, a necessary and sufficient condition of steerability has been derived from the relations between steerability and the joint measurability problem^31^. In the following, we study the steerability of any arbitrary given two-qubit states. We present analytical steerability conditions for classes of two-qubit *X*-state.

## Results

### Steerability of two-qubit states

First, based on the jointly measurable condition (1) of ${\{{O}_{\kappa }({x}_{i},{\overrightarrow{g}}_{i})\}}_{\kappa ,i}$ for the two-setting steering scenario, we define the steerability of two-qubit states ${\rho }_{AB}$ by the following

2 $$S=\,{\rm{\max }}\,\{\mathop{{\rm{\max }}}\limits_{{\alpha }_{i},{\beta }_{i}}({S}_{1}-{S}_{2}\mathrm{),0\},}$$

where ${S}_{1}=\mathrm{(1}-{F}_{{x}_{0}}^{2}-{F}_{{x}_{1}}^{2}\mathrm{)(1}-\frac{{x}_{0}^{2}}{{F}_{{x}_{0}}^{2}}-\frac{{x}_{1}^{2}}{{F}_{{x}_{1}}^{2}})$, ${S}_{2}={({\overrightarrow{g}}_{0}\cdot {\overrightarrow{g}}_{1}-{x}_{0}{x}_{1})}^{2}$, and the maximization runs over all of the measurements ${{\rm{\Pi }}}_{\kappa |{\overrightarrow{n}}_{i}}$, namely, over the parameters ${\alpha }_{i}$ and ${\beta }_{i}$, $i=\mathrm{0,1}$. It is obvious that *S* lies between 0 and 1 and ${\rho }_{AB}$ is steerable if and only if $S\, > \,0$.

For general two-qubit states, a global search can be used to obtain the global minimum values of *S*. The Matlab code is supplied in the supplementary material.

Due to the relationship between the joint measurements and steerability, local hidden states ${\tilde{\rho }}_{\kappa |{\overrightarrow{n}}_{i}}$ are represented as $\sqrt{{\rho }_{B}}{G}_{\mu v}\sqrt{{\rho }_{B}}$ $(\mu =\pm \mathrm{1,}v=\pm \mathrm{1),}$ where ${G}_{\mu v}=\frac{1}{4}\mathrm{(1}+\mu {x}_{0}+v{x}_{1}+\mu vZ+(\mu v\overrightarrow{z}+\mu {\overrightarrow{g}}_{0}+v{\overrightarrow{g}}_{1})\overrightarrow{\sigma })$ which are all possible sets of four measurements satisfying the marginal constraints for any two jointly measurable observables ${\{{O}_{\kappa }({x}_{i},{\overrightarrow{g}}_{i})\}}_{\kappa ,i}$ ^35–37^. The steering radius $R({\rho }_{AB})$ ^24^ can be calculated by optimizing $\overrightarrow{z}$ and *Z*.

In the following, we analytically calculate the steerability *S* for some *X*-states ${\rho }_{X}$. We define a class of two-qubit X-states to be zero-states ${\rho }_{zero}$ if the *X*-states ${\rho }_{X}$ satisfy the condition that the maximum points (stationary points) of *S*_1_ belong to the zero points of *S*_2_ with respect to the measurement parameters *α*_*i*_ and ${\beta }_{i},(i=\mathrm{1,2)}$.

For any two-qubit X-state, ${\rho }_{X}=\frac{1}{4}({{I}}_{4}+{a}_{3}{\sigma }_{3}\otimes {{I}}_{2}+{b}_{3}{{I}}_{2}\otimes {\sigma }_{3}+\sum _{i}^{3}{c}_{i}{\sigma }_{i}\otimes {\sigma }_{i})$, we have $U={\rm{Diag}}\{{u}_{1},{u}_{2},{u}_{3}\},$ $V\,=\,\mathrm{[0,}\,\mathrm{0,}\,{t}_{3}]$, where ${u}_{1}={c}_{1}/\sqrt{1-{b}_{3}^{2}}$, ${u}_{2}={c}_{2}/\sqrt{1-{b}_{3}^{2}}$, ${u}_{3}=({a}_{3}{b}_{3}-{c}_{3})/(-1+{b}_{3}^{2})$ and ${t}_{3}=({a}_{3}-{b}_{3}{c}_{3}\mathrm{)/}$ $\mathrm{(1}-{b}_{3}^{2}\mathrm{).}$We obtain the following results:

#### **Theorem**

. For the zero-states ${\rho }_{zero}$, the analytical formula of the steerability is given by

3 $$S=\,{\rm{\max }}\,\{{{\rm{\Delta }}}_{1},{{\rm{\Delta }}}_{2},{{\rm{\Delta }}}_{3},\,\mathrm{0\},}$$

where ${{\rm{\Delta }}}_{1}={u}_{1}^{2}+{u}_{2}^{2}-\mathrm{1,}$ ${{\rm{\Delta }}}_{2}=\frac{1}{2}[{u}_{1}^{2}({u}_{3}^{2}-{t}_{3}^{2})+{u}_{1}^{2}+{u}_{3}^{2}+{t}_{3}^{2}-1-\mathrm{(1}-{u}_{1}^{2})\sqrt{\mathrm{((1}-{t}_{3}{)}^{2}-{u}_{3}^{2}\mathrm{)((1}+{t}_{3}{)}^{2}-{u}_{3}^{2})}],$ ${{\rm{\Delta }}}_{3}=\frac{1}{2}[{u}_{2}^{2}({u}_{3}^{2}-{t}_{3}^{2})+{u}_{2}^{2}+{u}_{3}^{2}+{t}_{3}^{2}-1-\mathrm{(1}-{u}_{2}^{2})\times$ $\sqrt{\mathrm{((1}-{t}_{3}{)}^{2}-{u}_{3}^{2}\mathrm{)((1}+{t}_{3}{)}^{2}-{u}_{3}^{2})}\mathrm{].}$ When $S > \mathrm{0,}$ the optimal measurements that give rise to maximal *S* are ${\sigma }_{x}$ and ${\sigma }_{y}$ if ${{\rm{\Delta }}}_{1} > max\{{{\rm{\Delta }}}_{2},{{\rm{\Delta }}}_{3}\mathrm{,0\},}$ ${\sigma }_{x}$ and ${\sigma }_{z}$ if ${{\rm{\Delta }}}_{2} > \,{\rm{\max }}\,\{{{\rm{\Delta }}}_{1},{{\rm{\Delta }}}_{3},\,\mathrm{0\},}$ and ${\sigma }_{y}$ and ${\sigma }_{z}$ if ${{\rm{\Delta }}}_{3} > max\{{{\rm{\Delta }}}_{1},{{\rm{\Delta }}}_{2}\mathrm{,0\}}$.

The proof is given in the supplementary material.

It is obvious that any X-state with ${t}_{3}=0$ belongs to ${\rho }_{zero}$, e.g., $|\phi \rangle =a\mathrm{|00}\rangle +\sqrt{1-{a}^{2}}\mathrm{|11}\rangle$ $\mathrm{(0} < |a| < \mathrm{1)}$ and the Bell-diagonal state $\rho =\frac{1}{4}({\rm{I}}+{c}_{1}{\sigma }_{1}\otimes {\sigma }_{1}+{c}_{2}{\sigma }_{2}\otimes {\sigma }_{2}+{c}_{3}{\sigma }_{3}\otimes {\sigma }_{3})$ are all the zero states. For $|\phi \rangle ,$ we have $S=1$.

For the Bell-diagonal state, interestingly, the steerability $S$ is given by the non-locality characterized by the maximal violation of the CHSH inequality. Let ${ {\mathcal B} }_{CHSH}$ denote the Bell operator for the CHSH inequality^38^, ${ {\mathcal B} }_{CHSH}={A}_{1}\otimes {B}_{1}+{A}_{1}\otimes {B}_{2}+{A}_{2}\otimes {B}_{1}-{A}_{2}\otimes {B}_{2}$, where ${A}_{i}={\overrightarrow{a}}_{i}\cdot \overrightarrow{\sigma }$, ${B}_{i}={\overrightarrow{b}}_{i}\cdot \overrightarrow{\sigma }$, ${\overrightarrow{a}}_{i}$ and ${\overrightarrow{b}}_{i}$, $i=\mathrm{1,}\,2$, are unit vectors. Thus, the the maximal violation of the CHSH inequality is given by^39^

4 $$N=\mathop{{\rm{\max }}}\limits_{{ {\mathcal B} }_{{\mathscr{C}} {\mathcal H} {\mathscr{S}} {\mathcal H} }}|{\langle { {\mathcal B} }_{{\mathscr{C}} {\mathcal H} {\mathscr{S}} {\mathcal H} }\rangle }_{\rho }\mathrm{|=2}\sqrt{{\tau }_{1}+{\tau }_{2}},$$

where ${\tau }_{1}$ and ${\tau }_{2}$ are the two largest eigenvalues of the matrix ${T}^{\dagger }T$, $T$ is the matrix with entries ${T}_{\alpha \beta }=tr[\rho \,{\sigma }_{\alpha }\otimes {\sigma }_{\beta }]$, $\alpha ,\,\beta \,=\,\mathrm{1,2,3}$, $\dagger$ indicates transpose and conjugation. For the Bell-diagonal state, we have $N=$ $2\sqrt{{c}_{1}^{2}+{c}_{2}^{2}+{c}_{3}^{2}-\,{\rm{\min }}\,\{{c}_{1}^{2},{c}_{2}^{2},{c}_{3}^{2}\}}$. From (3), we find that the steerability of Bell-diagonal state is given by $S=\frac{{N}^{2}}{4}-1$.

For ${t}_{3}\ne 0$, we give the explicit conditions of the zero states in the supplementary material.

In the following, we present the maximum value of the steerability *S* for a given *N* of ${\rho }_{zero}$.

#### **Corollary 1**

: For zero-states ${\rho }_{zero}$ with given *N*, $0\le N\le 2$, we have $S\le \frac{N}{2}$. Moreover, $S=N\mathrm{/2}$ is attained when ${a}_{3}\,=\,1-{c}_{3}+{b}_{3},$ ${b}_{3}\to -\mathrm{1,}$ ${c}_{1}=\sqrt{\mathrm{(1}+{b}_{3})({c}_{3}-{b}_{3})},$ ${c}_{2}=-{c}_{1},$ i.e., ${\rho }_{zero}$ has the following form,

5 $${\rho }_{{X}_{0}}=(\begin{array}{cccc}\frac{1+{b}_{3}}{2} & 0 & 0 & \pm \frac{\sqrt{\mathrm{(1}+{b}_{3})({c}_{3}-{b}_{3})}}{2}\\ 0 & \frac{1-{c}_{3}}{2} & 0 & 0\\ 0 & 0 & 0 & 0\\ \pm \frac{\sqrt{\mathrm{(1}+{b}_{3})({c}_{3}-{b}_{3})}}{2} & 0 & 0 & \frac{{c}_{3}-{b}_{3}}{2}\end{array})\mathrm{.}$$

The following corollary gives the conditions at which we obtain the minimal value of *S* for a given *N*.

#### **Corollary 2**

: For zero-states ${\rho }_{zero}$ with given CHSH value *N*, *S* obtains the minimal value when ${a}_{3}\mathrm{=0}$ and ${b}_{3}\mathrm{=0}$ or $|{a}_{3}+{b}_{3}|=\sqrt{{\mathrm{(1}+{c}_{3})}^{2}-{({c}_{1}-{c}_{2})}^{2}}$ or $|{a}_{3}-{b}_{3}|=\sqrt{{\mathrm{(1}-{c}_{3})}^{2}-{({c}_{1}+{c}_{2})}^{2}}$.

**Figure 1:**
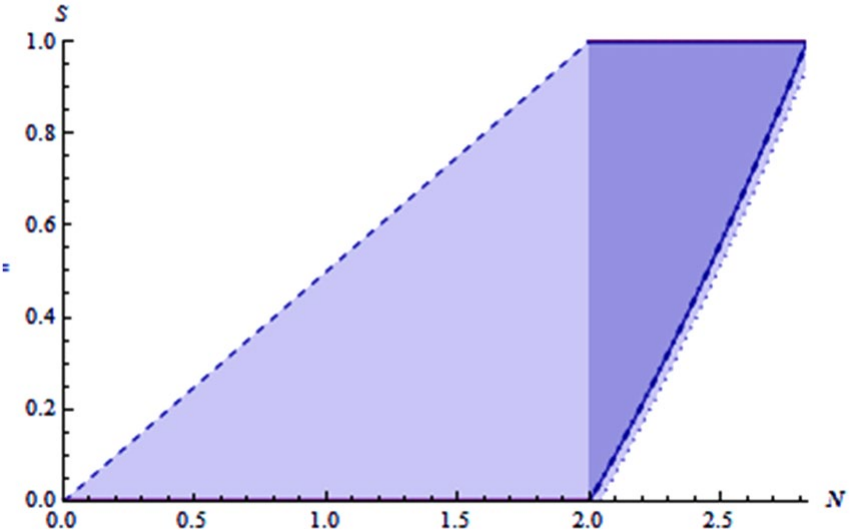
Regions of the values taken on by steerability *S* for given *N*.

The proofs of Corollary 1 and Corollary 2 are given in the supplementary material. In Fig. 1, we give a description for the boundaries of the steerability *S* for a given value of *N*. From Fig. 1, we observe that for any given *N* with $0\le N\le 2$, the lower bound of *S* is always 0 and the upper bound of *S* is always less than 2 (light blue), and for $N\mathrm{ > 2,}$ the lower bound of *S* is always greater than 0, and the upper bound of *S* is always 2 (dark blue).

For zero-states ${\rho }_{zero},$ the steering radius $R({\rho }_{zero})$ can be obtained when Alice measures her qubit along the directions ${\sigma }_{x}$ and ${\sigma }_{y},$ or ${\sigma }_{x}$ and ${\sigma }_{z},$ or ${\sigma }_{y}$ and ${\sigma }_{z}\mathrm{.}$ Indeed, from the construction of joint measurements^35^, when Alice measures her qubit along the directions of ${\sigma }_{x}$ and ${\sigma }_{z},$ the local hidden states can be expressed as follows

$$\frac{1}{2}({{I}}_{2}+\frac{{m}_{x}{\sigma }_{x}+{m}_{z}{\sigma }_{z}}{1+\mu {a}_{3}+v({b}_{3}{z}_{3}+Z)}),$$

where $${m}_{x}=\mu v({c}_{1}+\mu \sqrt{1-{b}_{3}^{2}}{z}_{1}),$$ $${m}_{z}={b}_{3}+\mu {c}_{3}+v({z}_{3}+{b}_{3}Z),$$ $$\mu =\pm \mathrm{1,}v=\pm 1.$$ Therefore

6 $$R({\rho }_{zero})=\,{\rm{\max }}\,\{r{({\rho }_{x})}_{xy},r{({\rho }_{x})}_{xz},r{({\rho }_{x})}_{yz}\},$$

where

$$\begin{array}{l}r{({\rho }_{zero})}_{xy}=\sqrt{{c}_{1}^{2}+{c}_{2}^{2}+{b}_{3}^{2}};\,r{({\rho }_{zero})}_{xz}=\mathop{{\rm{\min }}}\limits_{{z}_{1},{z}_{3},Z}\mathop{{\rm{\max }}}\limits_{\mu ,v}\sqrt{{r}_{\mu ,v}^{xz}};\,r{({\rho }_{zero})}_{yz}=\mathop{{\rm{\min }}}\limits_{{z}_{1},{z}_{3},Z}\mathop{{\rm{\max }}}\limits_{\mu ,v}\sqrt{{r}_{\mu ,v}^{yz}};\\ {r}_{\mu ,v}^{xz}=\tfrac{{({c}_{1}+\mu \sqrt{1-{b}_{3}^{2}}{z}_{1})}^{2}+{({b}_{3}+\mu {c}_{3}+v({z}_{3}+{b}_{3}Z))}^{2}}{{\mathrm{(1}+\mu {a}_{3}+v({b}_{3}{z}_{3}+Z))}^{2}};\,{r}_{\mu ,v}^{yz}=\tfrac{{({c}_{2}+\mu \sqrt{1-{b}_{3}^{2}}{z}_{1})}^{2}+{({b}_{3}+\mu {c}_{3}+v({z}_{3}+{b}_{3}Z))}^{2}}{{\mathrm{(1}+\mu {a}_{3}+v({b}_{3}{z}_{3}+Z))}^{2}}.\end{array}$$

It is not easy to calculate $r{({\rho }_{zero})}_{xz}$ and $r{({\rho }_{zero})}_{yz}$ analytically. We give the analytical results for $R({\rho }_{zero})$ for some special states in the following.

### Asymmetric two-setting EPR-steering

Different from Bell-nonlocality and quantum entanglement, EPR-steering has the asymmetric property of one-way EPR steering: Alice may steer Bob’s state but not vice versa. The demonstration of asymmetric steerability has practical implications in quantum communication networks^40^. Until now, only a few asymmetric steering states have been found^24,32–34^. In this work we present a class of asymmetric steering states of the form ${\rho }_{{X}_{0}}$ in (5).

**Figure 2:**
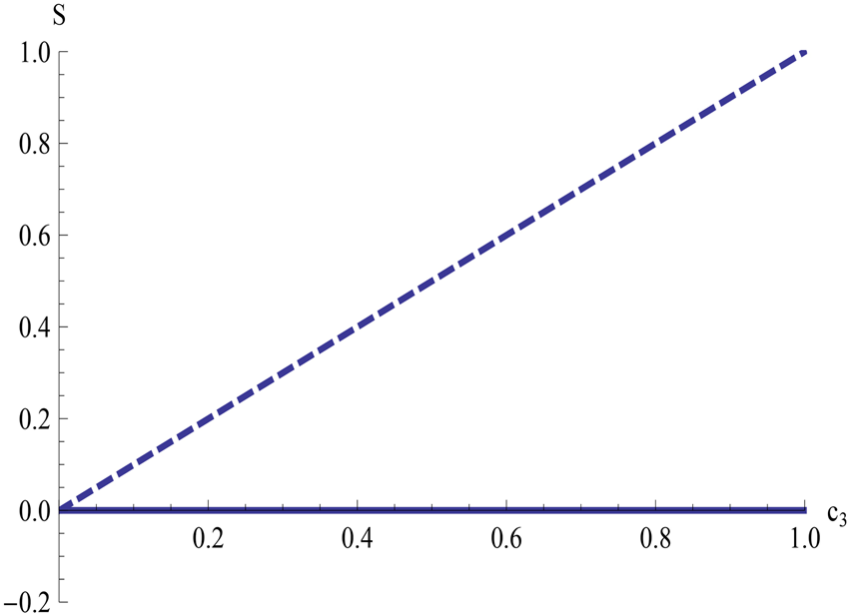
Steerability *S* versus ${c}_{3}$ for ${b}_{3}=-0.999$. The dashed line indicates Alice steering Bob’s state, and the solid line (horizontal coordinate) denotes Bob steering Alice’s state.

If Alice performs measurements on her qubit, the steerability is given by $S({\rho }_{{X}_{0}})=max\{\frac{2{c}_{3}-1-{b}_{3}}{1-{b}_{3}}\mathrm{,0\}}$ which approaches ${c}_{3}$ when ${b}_{3}$ approaches $-1$ and ${c}_{3} > 0$. If Bob performs measurements on his qubit, the related steerability is given by the following

$$S({\rho }_{{X}_{0}})=\,{\rm{\max }}\{\frac{\mathrm{(1}+{b}_{3})({b}_{3}+{c}_{3})}{{\mathrm{(2}+{b}_{3}-{c}_{3})}^{2}},0\}$$

which is equal to zero as long as $\mathrm{(1}+{b}_{3})({b}_{3}+{c}_{3})\le 0$. Therefore, when $0 < {c}_{3} < -{b}_{3}$ and ${b}_{3}\to -\mathrm{1,}$ Alice can always steer Bob’s state, but Bob can never steer Alice’s state (see Fig. 2 for the asymmetric EPR-steering for ${b}_{3}=-0.999$). We note that Alice can always steer Bob’s state, but Bob can not steer Alice’s state.

In the following subsection, we investigate the geometric features of the asymmetric steering state-${\rho }_{{x}_{0}}$ in terms of the steering ellipsoid^41^. The steering ellipsoid of ${\rho }_{{X}_{0}}$ when Alice performs POVMs is quite different from that when Bob performs POVMs. The centre of the steering ellipsoid ${\varepsilon }_{B}$ for Alice performing POVMs on her qubit is $\mathrm{(0,0,(}{b}_{3}-{a}_{3}{c}_{3}\mathrm{)/(1}-{a}_{3}^{2}))$, which goes to $\mathrm{(0,0,}-\mathrm{1)}$ when $b\to -\mathrm{1,}$ and the volume of the steering ellipsoid ${\varepsilon }_{B}$ is given as follows

$$\frac{4\pi }{3}\frac{|{c}_{1}{c}_{2}({c}_{3}-{a}_{3}{b}_{3})|}{{\mathrm{(1}-{a}_{3}^{2})}^{2}}=\frac{4\pi }{3}\frac{{\mathrm{(1}+{b}_{3})}^{2}}{{\mathrm{(2}-{c}_{3}+{b}_{3})}^{2}},$$

In this case the steering ellipsoid is tangent to the Bloch sphere. The centre of the steering ellipsoid ${\varepsilon }_{A}$ for Bob performing POVMs on his qubit is

$$(\mathrm{0,0,}\frac{{a}_{3}-{b}_{3}{c}_{3}}{1-{b}_{3}^{2}}=\mathrm{0,0,}\frac{1-{c}_{3}}{1-{b}_{3}}),$$

which goes to $\mathrm{(1}-{c}_{3}\mathrm{)/2}$ when ${b}_{3}\to -1$. The volume of the steering ellipsoid ${\varepsilon }_{A}$ is given by the following

$$\frac{4\pi }{3}\frac{|{c}_{1}{c}_{2}({c}_{3}-{a}_{3}{b}_{3})|}{{\mathrm{(1}-{b}_{3}^{2})}^{2}}=\frac{4\pi {({c}_{3}-{b}_{3})}^{2}}{\mathrm{3(1}-{b}_{3}{)}^{2}},$$

which goes to $\frac{\pi {\mathrm{(1}+{c}_{3})}^{2}}{3}$ when ${b}_{3}\to -1$. The steering ellipsoid is also tangent to the Bloch sphere. In this case the ellipsoid shows some peculiar features, i.e., when ${b}_{3}\to -1$ and ${c}_{3}\to 0$, the ellipsoid ${\varepsilon }_{B}$ is nearly $0$, but Alice can still steer Bob; however, when ${b}_{3}\to -1$ and ${c}_{3}\to -{b}_{3}$, the ellipsoid ${\varepsilon }_{A}$ is almost the entire Bloch sphere, but Bob can not steer Alice.

As a special case of ${\rho }_{{X}_{0}},$ we take ${a}_{3}=1-2\eta \mathrm{(1}-\chi ),$ ${b}_{3}=2\eta \chi -\mathrm{1,}$ ${c}_{3}=2\eta -\mathrm{1,}$ ${c}_{1}=-{c}_{2}=-2\eta \sqrt{\chi \mathrm{(1}-\chi )}$. The state has the following form,

7 $${W}_{\eta }^{\chi }=(\begin{array}{cccc}\eta \chi  & 0 & 0 & -\eta \sqrt{\chi \mathrm{(1}-\chi )}\\ 0 & 1-\eta  & 0 & 0\\ 0 & 0 & 0 & 0\\ -\eta \sqrt{\chi \mathrm{(1}-\chi )} & 0 & 0 & \eta \mathrm{(1}-\chi )\end{array})\mathrm{.}$$

From the theorem, we obtain the following when Alice measures her qubit,

$$\begin{array}{ccc}S({W}_{\eta }^{\chi }) & = & {\rm{\max }}\{\frac{1+\eta (-2+\chi )}{-1+\eta \chi },\frac{\eta \mathrm{(1}+\eta (-2+\chi ))(-1+\chi )}{{\mathrm{(1}-\eta \chi )}^{2}}\mathrm{,0}\}\mathrm{.}\end{array}$$

The sufficient and necessary condition in the two-setting steering scenario is $\eta \, > \,\mathrm{1/(2}-\chi )$ for Alice to steer Bob’s state. The corresponding optimal measurements are ${\sigma }_{x}$ and ${\sigma }_{y}\mathrm{.}$

If Bob measures his qubit, the steerability is given by the following

$$S({W}_{\eta }^{\chi })=\,{\rm{\max }}\{\frac{\eta \chi (-1+\eta +\eta \chi )}{{\mathrm{(1}+\eta (-1+\chi ))}^{2}},\frac{-1+\eta +\eta \chi }{1+\eta (-1+\chi )}\mathrm{,0}\}\mathrm{.}$$

**Figure 3:**
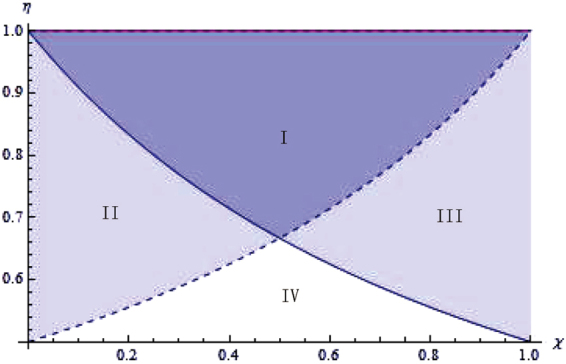
Parameter region for which Alice (Bob) can steer Bob’s (Alice’s) state for the state ${W}_{\eta }^{\chi }$. In region I, Alice can steer Bob’s state, and Bob can also steer Alice’s state. In region II (III), Alice (Bob) can steer Bob’s (Alice’s) state, but Bob (Alice) can not steer Alice’s (Bob’s) state. In region IV, Alice can not steer Bob’s state, and Bob can not steer Alice’s state.

**Figure 4:**
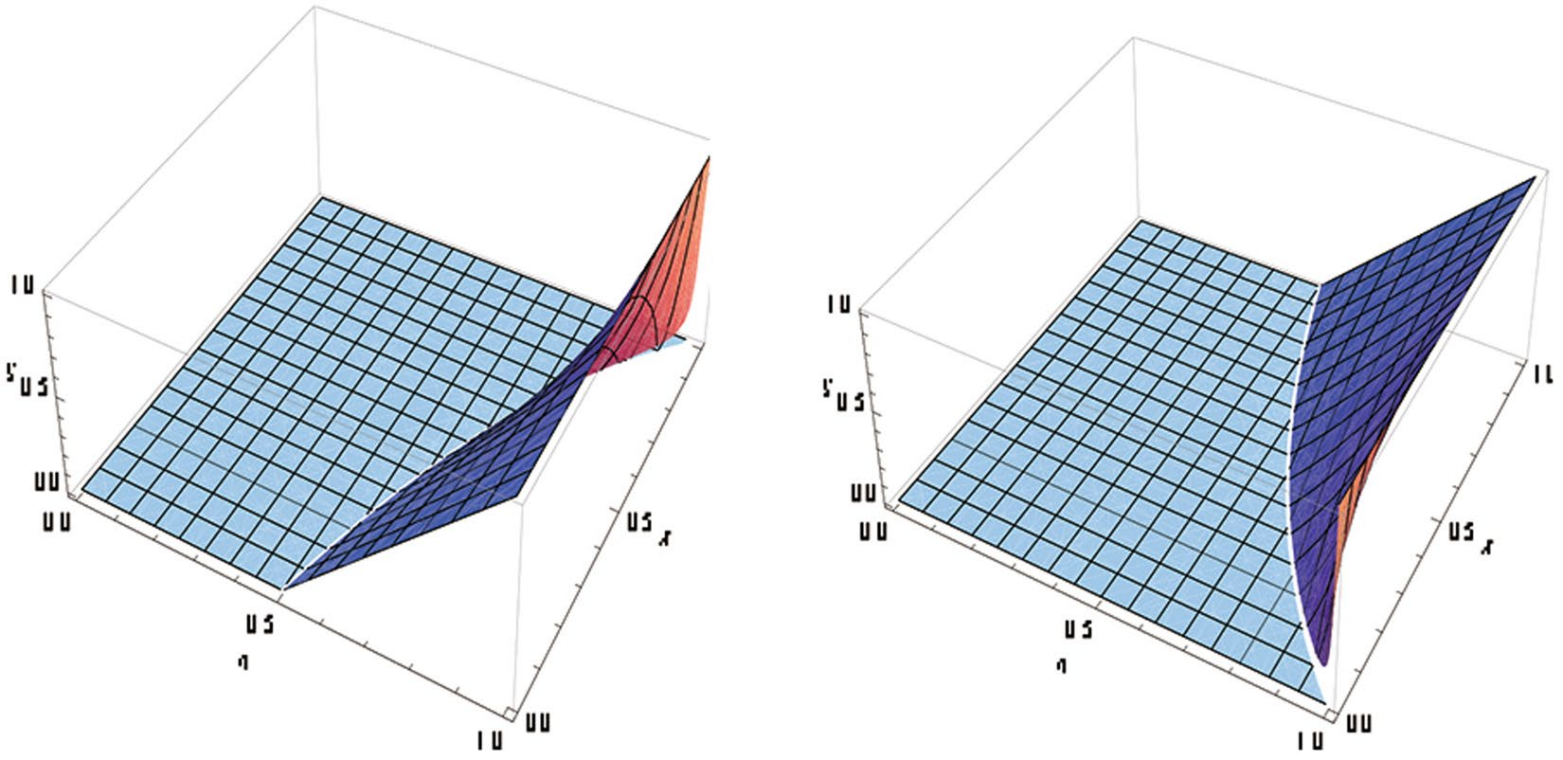
The left figure(the right figure): $S({W}_{\eta }^{\chi })$ when Alice (Bob) measures her (his) qubit.

The sufficient and necessary condition for Bob to steer Alice’s state is $\eta \mathrm{ > 1/(1}+\chi )$. The related optimal measurements are ${\sigma }_{x}$ and ${\sigma }_{y}\mathrm{.}$ The asymmetric property in quantum steering given by this example is shown in Figs 3 and 4. The steering radius is $\sqrt{1-4\eta \chi \mathrm{(1}-\eta \mathrm{(2}-\chi ))}$ when Alice measures her qubit, and $\sqrt{1-4\eta \mathrm{(1}-\chi \mathrm{)(1}-\eta -\eta \chi )}$ when Bob measures his qubit.

As another example showing the asymmetry of quantum steering, we consider the state ${W}_{V}^{\theta }$ ^24^,

8 $${W}_{V}^{\theta }=V|{\psi }_{1}\rangle \langle {\psi }_{1}|+\mathrm{(1}-V)|{\psi }_{2}\rangle \langle {\psi }_{2}|,$$

where $|{\psi }_{1}\rangle =\,\cos \,\theta \mathrm{|00}\rangle +\,\sin \,\theta \mathrm{|11}\rangle ,$ $|{\psi }_{2}\rangle =\,\cos \,\theta \mathrm{|10}\rangle +\,\sin \,\theta \mathrm{|01}\rangle ,$ $\theta \in \mathrm{(0,}\,\pi \mathrm{/2),}\,V\in \mathrm{[0,}\,\mathrm{1/2)}\cup \mathrm{(1/2,}\,\mathrm{1]}$. ${W}_{V}^{\theta }$ is a zero state. From our theorem, we know that when Alice performs measurements on her qubit, $S({W}_{V}^{\theta })={\mathrm{(1}-2V)}^{2}$. The optimal measurements are ${\sigma }_{x},$ ${\sigma }_{y}$ or ${\sigma }_{x},$ ${\sigma }_{z}\mathrm{.}$ This state is always steerable for Alice except when $V\mathrm{=1/2}$.

When Bob performs two projective measurements on his qubit, we have the following

9 $$\begin{array}{ll}S({W}_{V}^{\theta })\,= & {\rm{\max }}\{\frac{{\mathrm{(1}-2V)}^{2}-{\cos }^{2}2\theta }{1-{\mathrm{(1}-2V)}^{2}{\cos }^{2}2\theta },\frac{\sin \,2{\theta }^{2}\mathrm{((1}-2V{)}^{2}-{\cos }^{2}2\theta )}{{\mathrm{(1}-{\mathrm{(1}-2V)}^{2}{\cos }^{2}2\theta )}^{2}}\mathrm{,0}\}\mathrm{.}\end{array}$$

**Figure 5:**
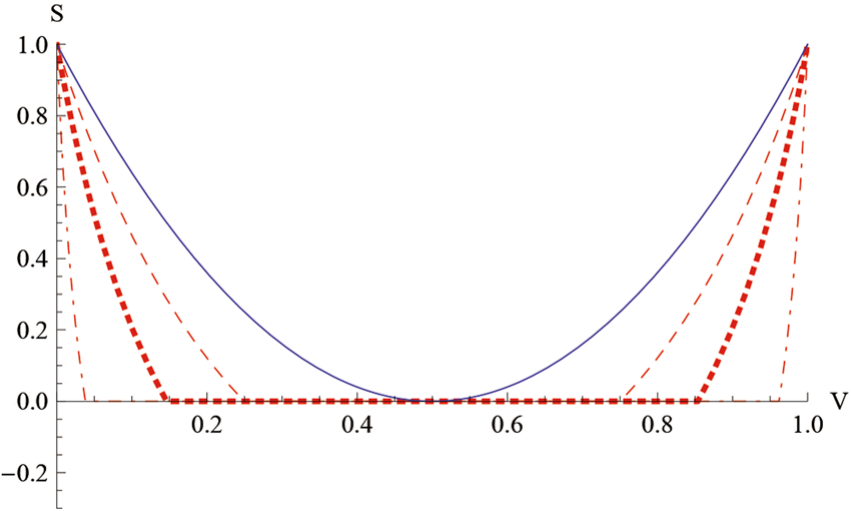
$S({W}_{V}^{\theta })$ versus *θ*: the blue solid line denotes when Alice measures her qubit, and the red dashed line $(\theta =\frac{\pi }{6})$, red dotted line $(\theta =\frac{\pi }{8})$, and red dot-dashed line $(\theta =\frac{\pi }{16})$ indicate when Bob measures his qubit.

The sufficient and necessary condition in the two-setting steering scenario for Bob to steer Alice’s state is $|cos2\theta \mathrm{| < |2}V-\mathrm{1|}$, with the optimal measurements ${\sigma }_{x}$ and ${\sigma }_{y}$. For ${W}_{V}^{\theta },$ the corresponding steering radius is $\sqrt{1+{\mathrm{(1}-2V)}^{2}{\sin }^{2}2\theta }$ when Alice measures her qubit, and $\sqrt{{\mathrm{(1}-2V)}^{2}+{\sin }^{2}2\theta }$ when Bob measures his qubit. From Fig. 5 we observe that Alice can always steer Bob’s state except when $V\mathrm{=1/2,}$ but Bob can steer Alice’s state only for some *V* depending on *θ*.

From our theorem, the analytical results of steerability can be obtained for more detailed zero states, and the asymmetric property of steering can be readily studied. In the following, we give two examples of symmetric two-setting EPR-steering.

#### Example 1

. The two-qubit nonmaximally entangled state mixed with colour noise,

$${\rho }_{{cn}}=V|\psi (\theta )\rangle \langle \psi (\theta )|+\frac{1-V}{2}\mathrm{(|00}\rangle \langle \mathrm{00|}+\mathrm{|11}\rangle \langle \mathrm{11|),}$$

where $|\psi (\theta )\rangle =\,\cos \,\theta \mathrm{|00}\rangle +\,\sin \,\theta \mathrm{|11}\rangle$, $\theta \in \mathrm{(0,}\pi \mathrm{/2)}$, $V\in \mathrm{(0,1]}$. The steerability is given by $S({\rho }_{{\rm{c}}n})={V}^{2}{\sin }^{2}2\theta \mathrm{/}$ $\mathrm{(1}-{V}^{2}\,\cos \,2{\theta }^{2})$. Therefore, ${\rho }_{{\rm{c}}n}$ is steerable if and only if $V{\rm{s}}{\rm{i}}{\rm{n}}2\theta \ne 0.$

#### Example 2

. The generalized isotropic state, ${\rho }_{gi}=V|\psi (\theta )\rangle \langle \psi (\theta )|+\mathrm{(1}-V){\rm{I}}\mathrm{/4}$, where $|\psi (\theta )\rangle =\,\cos \,\theta \mathrm{|00}\rangle +$ $\sin \,\theta \mathrm{|11}\rangle$, $\theta \in \mathrm{(0,}\pi \mathrm{/2)}$, $V\in \mathrm{(0,1]}$. The state reduces to the usual isotropic state when $\theta =\pi \mathrm{/4}$. According to our theorem, we obtain the analytical steerability of ${\rho }_{gi}$,

$$S({\rho }_{gi})=\tfrac{1-{V}^{2}{\cos }^{2}4\theta +\mathrm{(1}-V)\sqrt{{\mathrm{(1}+V)}^{2}-4{V}^{2}{\cos }^{2}2\theta }}{\mathrm{4(1}-{V}^{2}{\cos }^{2}2\theta )}\times \tfrac{{V}^{2}\mathrm{(1}+2{\sin }^{2}2\theta )-1-\mathrm{(1}-V)\sqrt{{\mathrm{(1}+V)}^{2}-4{V}^{2}{\cos }^{2}2\theta }}{1-{V}^{2}{\cos }^{2}2\theta }\mathrm{.}$$

Hence, the sufficient and necessary condition of steerability is $1+\mathrm{(1}-V)\sqrt{{\mathrm{(1}+V)}^{2}-4{V}^{2}{\cos }^{2}2\theta } <$ ${V}^{2}\mathrm{(1}+2{\sin }^{2}2\theta \mathrm{).}$

## Discussion

Based on the one-to-one correspondence between EPR-steering and joint measurability, we have investigated the steerability for any two-qubit system in the two-setting measurement scenario. The steerability we introduced is invariant under local unitary operations. The analytical formula for steerability has been derived for a class of X-states, and the sufficient and necessary conditions for two-setting EPR-steering have been presented. For general two-qubit states, it has been shown that the lower and upper bounds of steerability are explicitly connected to the non-locality of the states given by the CHSH values of maximal violation. Moreover, we have also presented a class of asymmetric steering states by investigating steerability with respect to the measurements from Alice’s and Bob’s sides. Our strategy might also be used to study the quantification of steerability for multi-setting scenarios, in particular, for three-setting scenarios for which the joint measurability problem of three qubit observables has already been investigated^42,43^. Our method might also be used in continuous variable steering, temporal and channel steering, for which the steerability of the state assemblages or the instrument assemblages can be connected to the incompatibility problems of the quantum measurement assemblages^44,45^. Hence, the steerability of the quantum states or the quantum channels might also be studied based on the corresponding measurement incompatibility problems.

## Electronic supplementary material

Supplementary information
