# Supplementary material for: Quantum steerability based on joint measurability
Source: Sci Rep. 2017 Nov 17;7:15822. doi: 10.1038/s41598-017-15910-8 (PMC5693965; doi:10.1038/s41598-017-15910-8)
Supplement: Supplementary file 1 — Supplementary information [file 41598_2017_15910_MOESM1_ESM.pdf]

Supplementary for quantum steerability based on joint measurability

Zhihua Chen[1], **Xiangjun Ye**[2,3], **and Shao-Ming Fei**[4,5,*]

[1]Department of Mathematics, College of Science, Zhejiang University of Technology, Hangzhou 310023, China
[2]Key Laboratory of Quantum Information, University of Science and Technology of China, CAS, Hefei 230026, China
[3]Synergetic Innovation Center of Quantum Information and Quantum Physics, University of Science and Technology of China, Hefei, 230026, China
[4]School of Mathematical Sciences, Capital Normal University, Beijing 100048, China
[5]Max-Planck-Institute for Mathematics in the Sciences, 04103 Leipzig, Germany
[*]Corresponding-author: feishm@cnu.edu.cn

0.1 Proof of the Theorem

Denote $\delta=\sqrt{\frac{((1+x_1)^2-g_1^2)((1-x_1)^2-g_1^2)}{((1+x_0)^2-g_0^2)((1-x_0)^2-g_0^2)}}$, $\delta_1=1-x_1^2+g_1^2+\delta(1+x_0^2-g_0^2)$, $\delta_2=1+x_1^2-g_1^2+\delta(1-x_0^2+g_0^2)$, $\delta_3=\frac{\delta_2}{\delta}$ and $\delta_4=\frac{\delta_1}{\delta}$. To calculate the term $\max\limits_{\alpha_i,\beta_i} S_1$ of steerability, we compute the derivations of S_1 with respect to the variables α_i and β_i, $i=1,2$,

$$\begin{cases}\frac{\partial S_1}{\partial\alpha_1}= & \sin\alpha_1\cos\alpha_1[\delta_1u_1^2\cos^2\beta_1+\delta_1u_2^2\sin^2\beta_1-\delta_1u_3^2-\delta_2t_3^2], \qquad \frac{\partial S_1}{\partial\beta_1}=\delta_1\sin^2\alpha_1\sin\beta_1\cos\beta_1(u_2^2-u_1^2),\\ \frac{\partial S_1}{\partial\alpha_2}= & \sin\alpha_2\cos\alpha_2[\delta_3u_1^2\cos^2\beta_2+\delta_3u_2^2\sin^2\beta_2-\delta_3u_3^2-\delta_4t_3^2], \qquad \frac{\partial S_1}{\partial\beta_2}=\delta_3\sin^2\alpha_2\sin\beta_2\cos\beta_2(u_2^2-u_1^2).\end{cases}$$

From $\frac{\partial S_1}{\partial\alpha_1}=\frac{\partial S_1}{\partial\beta_1}=\frac{\partial S_1}{\partial\alpha_2}=\frac{\partial S_1}{\partial\beta_2}=0$, we have the following solutions,

$$\begin{cases}\sin\alpha_1\cos\alpha_1=0 \quad \text{or} \quad \Delta=0,\\ \sin^2\alpha_1\sin\beta_1\cos\beta_1=0,\\ \sin\alpha_2\cos\alpha_2=0 \quad \text{or} \quad \Omega=0,\\ \sin^2\alpha_2\sin\beta_2\cos\beta_2=0,\end{cases}$$

where $\Delta=\delta_1(u_1^2\cos^2\beta_1+u_2^2\sin^2\beta_1-u_3^2)-\delta_2t_3^2$ and $\Omega=\delta_3(u_1^2\cos^2\beta_2+u_2^2\sin^2\beta_2-u_3^2)-\delta_4t_3^2$. Therefore we have either

$$\begin{cases}\sin\alpha_1\cos\alpha_1=0,\\ \sin^2\alpha_1\sin\beta_1\cos\beta_1=0,\\ \sin\alpha_2\cos\alpha_2=0,\\ \sin^2\alpha_2\sin\beta_2\cos\beta_2=0,\end{cases}\tag{1}$$

or

$$\begin{cases}\sin\alpha_1\cos\alpha_1=0,\\ \sin^2\alpha_1\sin\beta_1\cos\beta_1=0,\\ \sin\alpha_2\cos\alpha_2\neq0 \quad \text{but} \quad \Omega=0,\\ \sin^2\alpha_2\sin\beta_2\cos\beta_2=0,\end{cases}\tag{2}$$

or

$$\begin{cases} \sin\alpha_1\cos\alpha_1 \neq 0 \quad \text{but} \quad \Delta = 0, \\ \sin^2\alpha_1 \sin\beta_1\cos\beta_1 = 0, \\ \sin\alpha_2\cos\alpha_2 = 0, \\ \sin^2\alpha_2 \sin\beta_2\cos\beta_2 = 0, \end{cases} \tag{3}$$

or

$$\begin{cases} \sin\alpha_1\cos\alpha_1 \neq 0 \quad \text{but} \quad \Delta = 0, \\ \sin^2\alpha_1 \sin\beta_1\cos\beta_1 = 0, \\ \sin\alpha_2\cos\alpha_2 \neq 0 \quad \text{but} \quad \Omega = 0, \\ \sin^2\alpha_2 \sin\beta_2\cos\beta_2 = 0. \end{cases} \tag{4}$$

Actually, (2) is equivalent to (3). Hence, we only need to consider (1), (2) and (4). From (2), we have

$$\begin{cases} \cos\alpha_1 = 0, \\ \sin\beta_1\cos\beta_1 = 0, \\ \Omega = 0, \\ \sin\beta_2\cos\beta_2 = 0, \end{cases} \quad \text{or} \quad \begin{cases} \sin\alpha_1 = 0, \\ \Omega = 0, \\ \sin\beta_2\cos\beta_2 = 0, \end{cases} \tag{5}$$

which gives rise to

$$\begin{cases} \alpha_1 = \frac{\pi}{2}, \\ \beta_1 = \frac{(i-1)\pi}{2}, \\ \Omega = 0, \\ \beta_2 = \frac{(j-1)\pi}{2}, \end{cases} \quad \text{or} \quad \begin{cases} \alpha_1 = 0, \\ \Omega = 0, \\ \beta_2 = \frac{(j-1)\pi}{2}. \end{cases} \tag{6}$$

(4) is equivalent to

$$\begin{cases} \Delta = 0, \\ \sin\beta_1\cos\beta_1 = 0, \\ \Omega = 0, \\ \sin\beta_2\cos\beta_2 = 0, \end{cases} \Longrightarrow \begin{cases} \Delta = 0, \\ \beta_1 = \frac{(i-1)\pi}{2}, \\ \Omega = 0, \\ \beta_2 = \frac{(j-1)\pi}{2}. \end{cases} \tag{7}$$

Here $i = 1,2$ and $j = 1,2$. Form (6), given $\alpha_1 = 0$, $\beta_2 = \frac{(j-1)\pi}{2}$ or $\alpha_1 = \frac{\pi}{2}$, $\beta_1 = \frac{(i-1)\pi}{2}$, $\beta_2 = \frac{(j-1)\pi}{2}$, $\Omega = 0$ is an equation satisfied by α_2. From (7), given $\beta_1 = \frac{(i-1)\pi}{2}, \beta_2 = \frac{(j-1)\pi}{2}$, then $\Delta = 0$ and $\Omega = 0$ are equations satisfied by the variables α_1 and α_2. Hence we have the following conditions:

(I) For $\alpha_1 = \frac{\pi}{2}$, $\beta_1 = \frac{(i-1)\pi}{2}$ and $\beta_2 = \frac{(j-1)\pi}{2}$, if the equation $\Omega = 0$

(a) does not have a solution, or

(b) only has the solution $\alpha_2 = \frac{m\pi}{2}$ $(m = 0,1)$, or

(c) has the solutions $\alpha_2 = \alpha_2^0 \neq \frac{m\pi}{2}$, but this solution $\alpha_2 = \alpha_2^0$, together with $\alpha_1 = \frac{\pi}{2}$, $\beta_1 = \frac{(i-1)\pi}{2}$ and $\beta_2 = \frac{(j-1)\pi}{2}$, are not the maximum points of S_1.

(II) For $\alpha_1 = 0$, $\beta_2 = \frac{(j-1)\pi}{2}$, if the equation $\Omega = 0$

(a) does not have a solution, or

(b) only has the solutions $\alpha_2 = \frac{m\pi}{2}$, $m = 0,1$, or

(c) has the solutions $\alpha_2 = \alpha_2^1 \neq \frac{m\pi}{2}$, but $\alpha_2 = \alpha_2^1$, together with $\alpha_1 = 0$, $\beta_2 = \frac{(j-1)\pi}{2}$, are not the maximum points of S_1.

(III) For $\beta_1 = \frac{(i-1)\pi}{2}$ and $\beta_2 = \frac{(j-1)\pi}{2}$, the equations $\Delta = 0$ and $\Omega = 0$ are satisfied simultaneously if and only if $\alpha_1 = \frac{m\pi}{2}$, $\alpha_2 = \frac{n\pi}{2}$, $m = 0,1, n = 0,1$.

It is obvious that if ρ_X satisfies all the conditions (I) to (III), the candidates of the maximal points of S_1 are $\alpha_1=\frac{\pi}{2}$, $\alpha_2=0$, $\beta_1=0$ or $\alpha_1=\frac{\pi}{2}$, $\alpha_2=0$, $\beta_1=\frac{\pi}{2}$ or $\alpha_1=0$, $\alpha_2=\frac{\pi}{2}$, $\beta_2=0$ or $\alpha_1=0$, $\alpha_2=\frac{\pi}{2}$, $\beta_2=\frac{\pi}{2}$ or $\alpha_1=0$, $\alpha_2=0$ or $\alpha_1=\frac{\pi}{2}$, $\alpha_2=\frac{\pi}{2}$, $\beta_1=0$, $\beta_2=\frac{\pi}{2}$ or $\alpha_1=\frac{\pi}{2}$, $\alpha_2=\frac{\pi}{2}$, $\beta_1=\frac{\pi}{2}$, $\beta_2=0$, therefore the maximum points of S_1 are all the zero points of S_2, i.e. the states satisfying (I)-(III) are zero-states ρ_{zero}. We do not need to consider the case $\alpha_1=\alpha_2=0$, since when $\alpha_1=\alpha_2=0$, $S_1-S_2\leqslant 0$. Therefore, $S=\max\{\Delta_1,\Delta_2,\Delta_3,0\}$. □

0.2 Conditions of ρ_{zero} for X-state

For any given two-qubit X-state, it is difficult to check if the state belongs to zero-state or not. Here we study further the conditions that a X-state needs to satisfy to be a zero-state ρ_{zero}. In the following we denote $cond_{zero}$ the conditions such that ρ_X satisfying $cond_{zero}$ is a zero state.

We have already classified the problem by conditions (I)-(III). For conditions (I): $\alpha_1=\frac{\pi}{2}$, $\beta_1=\frac{(i-1)\pi}{2}$ and $\beta_2=\frac{(j-1)\pi}{2}(i,j=1,2)$, $\Omega=0$ is actually an equation satisfied by $\cos\alpha_2$. We can prove that the following conditions are equivalent to (I),

1a1).

$$\begin{cases} u_j^2<u_3^2 \quad \text{or} \quad [(u_i^2(u_j^2-u_3^2+t_3^2)-t_3^2)(u_j^2-u_3^2)][u_j^2(u_j^2-u_3^2+t_3^2)-u_j^2+u_3^2]<0, i=1,j=1\\ u_j^2<u_3^2 \quad \text{or} \quad [(u_i^2(u_j^2-u_3^2+t_3^2)-t_3^2)(u_j^2-u_3^2)][u_j^2(u_j^2-u_3^2+t_3^2)-u_j^2+u_3^2]<0, i=1,j=2\\ u_j^2<u_3^2 \quad \text{or} \quad [(u_i^2(u_j^2-u_3^2+t_3^2)-t_3^2)(u_j^2-u_3^2)][u_j^2(u_j^2-u_3^2+t_3^2)-u_j^2+u_3^2]<0, i=2,j=1\\ u_j^2<u_3^2 \quad \text{or} \quad [(u_i^2(u_j^2-u_3^2+t_3^2)-t_3^2)(u_j^2-u_3^2)][u_j^2(u_j^2-u_3^2+t_3^2)-u_j^2+u_3^2]<0, i=2,j=2 \end{cases}$$

1a2). if the conditions in 1a1) are not satisfied, that is, at least one of the four inequalities in 1a1) is not satisfied, i.e., for i and j which satisfy $u_j^2\geqslant u_3^2$ and $[(u_i^2(u_j^2-u_3^2+t_3^2)-t_3^2)(u_j^2-u_3^2)][u_j^2(u_j^2-u_3^2+t_3^2)-u_j^2+u_3^2]\geqslant 0$, we obtain the following

$$\begin{cases} \dfrac{(u_3^2-u_j^2+t_3^2+u_j^2(u_j^2-u_3^2+t_3^2))}{(u_j^2-u_3^2+t_3^2)^2}+\dfrac{|t_3||u_i^2(u_j^2-u_3^2+t_3^2)+u_j^2-u_3^2-t_3^2|}{(u_j^2-u_3^2+t_3^2)^2}\sqrt{\dfrac{u_3^2-u_j^2+u_j^2(u_j^2-u_3^2+t_3^2)}{(u_j^2-u_3^2)(u_i^2(u_j^2-u_3^2+t_3^2)-t_3^2)}}>1\\ \dfrac{(u_3^2-u_j^2+t_3^2+u_j^2(u_j^2-u_3^2+t_3^2))}{(u_j^2-u_3^2+t_3^2)^2}-\dfrac{|t_3||u_i^2(u_j^2-u_3^2+t_3^2)+u_j^2-u_3^2-t_3^2|}{(u_j^2-u_3^2+t_3^2)^2}\sqrt{\dfrac{u_3^2-u_j^2+u_j^2(u_j^2-u_3^2+t_3^2)}{(u_j^2-u_3^2)(u_i^2(u_j^2-u_3^2+t_3^2)-t_3^2)}}<0. \end{cases}$$

1a3). if the conditions in 1a1) and 1a2) are not satisfied, i.e., for i and j which satisfy $u_j^2\geqslant u_3^2$ and $[(u_i^2(u_j^2-u_3^2+t_3^2)-t_3^2)(u_j^2-u_3^2)][u_j^2(u_j^2-u_3^2+t_3^2)-u_j^2+u_3^2]\geqslant 0$, we obtain the following

$$\begin{cases} \cos^2\alpha_2=\dfrac{(u_3^2-u_j^2+t_3^2+u_j^2(u_j^2-u_3^2+t_3^2))}{(u_j^2-u_3^2+t_3^2)^2}+\dfrac{|t_3||u_i^2(u_j^2-u_3^2+t_3^2)+u_j^2-u_3^2-t_3^2|}{(u_j^2-u_3^2+t_3^2)^2}\sqrt{\dfrac{u_3^2-u_j^2+u_j^2(u_j^2-u_3^2+t_3^2)}{(u_j^2-u_3^2)(u_i^2(u_j^2-u_3^2+t_3^2)-t_3^2)}}\leqslant 1\\ \dfrac{(-1+u_i^2)(2u_j^2-u_j^4-2u_3^2+u_3^4-2(1+u_3^2)t_3^2+t_3^4+(u_j^2-u_3^2+t_3^2)^2)(2\cos\alpha_2^2-1)}{2(1+u_i^2)(u_j^2-u_3^2)-2(1-u_i^2)t_3^2}<0 \end{cases}$$

or

$$\begin{cases} \cos^2\alpha_2=\dfrac{(u_3^2-u_j^2+t_3^2+u_j^2(u_j^2-u_3^2+t_3^2))}{(u_j^2-u_3^2+t_3^2)^2}-\dfrac{|t_3||u_i^2(u_j^2-u_3^2+t_3^2)+u_j^2-u_3^2-t_3^2|}{(u_j^2-u_3^2+t_3^2)^2}\sqrt{\dfrac{u_3^2-u_j^2+u_j^2(u_j^2-u_3^2+t_3^2)}{(u_j^2-u_3^2)(u_i^2(u_j^2-u_3^2+t_3^2)-t_3^2)}}\geqslant 0\\ \dfrac{(-1+u_i^2)(2u_j^2-u_j^4-2u_3^2+u_3^4-2(1+u_3^2)t_3^2+t_3^4+(u_j^2-u_3^2+t_3^2)^2)(2\cos\alpha_2^2-1)}{2(1+u_i^2)(u_j^2-u_3^2)-2(1-u_i^2)t_3^2}<0 \end{cases}$$

If ρ_X satisfies conditions 1a1) or 1a2) or 1a3), we obtain that $\Omega=0$ does not have solutions for $\alpha_1=\frac{\pi}{2}$, $\beta_1=\frac{(i-1)\pi}{2}$ and $\beta_2=\frac{(j-1)\pi}{2}$.

If both 1a1),1a2) and 1a3) are not satisfied, then

1b) for the i and j which satisfy $u_j^2 \geqslant u_3^2$ and $[(u_i^2(u_j^2-u_3^2+t_3^2)-t_3^2)(u_j^2-u_3^2)][u_j^2(u_j^2-u_3^2+t_3^2)-u_j^2+u_3^2] \geqslant 0$ we obtain the following

$$\begin{cases} \cos^2\alpha_2 = \dfrac{(u_3^2-u_j^2+t_3^2+u_j^2(u_j^2-u_3^2+t_3^2))}{(u_j^2-u_3^2+t_3^2)^2} + \dfrac{|t_3||u_i^2(u_j^2-u_3^2+t_3^2)+u_j^2-u_3^2-t_3^2|}{(u_j^2-u_3^2+t_3^2)^2}\sqrt{\dfrac{u_3^2-u_j^2+u_j^2(u_j^2-u_3^2+t_3^2)}{(u_j^2-u_3^2)(u_i^2(u_j^2-u_3^2+t_3^2)-t_3^2)}} = 1 \\ \dfrac{(-1+u_i^2)(2u_j^2-u_j^4-2u_3^2+u_3^4-2(1+u_3^2)t_3^2+t_3^4+(u_j^2-u_3^2+t_3^2)^2)}{2(1+u_i^2)(u_j^2-u_3^2)-2(1-u_i^2)t_3^2} \geqslant 0. \end{cases}$$

or

$$\begin{cases} \cos^2\alpha_2 = \dfrac{(u_3^2-u_j^2+t_3^2+u_j^2(u_j^2-u_3^2+t_3^2))}{(u_j^2-u_3^2+t_3^2)^2} - \dfrac{|t_3||u_i^2(u_j^2-u_3^2+t_3^2)+u_j^2-u_3^2-t_3^2|}{(u_j^2-u_3^2+t_3^2)^2}\sqrt{\dfrac{u_3^2-u_j^2+u_j^2(u_j^2-u_3^2+t_3^2)}{(u_j^2-u_3^2)(u_i^2(u_j^2-u_3^2+t_3^2)-t_3^2)}} = 0 \\ \dfrac{(-1+u_i^2)(2u_j^2-u_j^4-2u_3^2+u_3^4-2(1+u_3^2)t_3^2+t_3^4-(u_j^2-u_3^2+t_3^2)^2)}{2(1+u_i^2)(u_j^2-u_3^2)-2(1-u_i^2)t_3^2} \geqslant 0. \end{cases}$$

i.e., for $\alpha_1 = \frac{\pi}{2}$, $\beta_1 = \frac{(i-1)\pi}{2}$ and $\beta_2 = \frac{(j-1)\pi}{2}$, $\Omega = 0$ has the solution $\alpha_2 = \frac{m\pi}{2}$ ($m = 0$ or 1).

1c) for the i and j which satisfy $u_j^2 \geqslant u_3^2$ and $[(u_i^2(u_j^2-u_3^2+t_3^2)-t_3^2)(u_j^2-u_3^2)][u_j^2(u_j^2-u_3^2+t_3^2)-u_j^2+u_3^2] \geqslant 0$, we obtain the following

$$\begin{cases} \cos^2\alpha_2 = \dfrac{(u_3^2-u_j^2+t_3^2+u_j^2(u_j^2-u_3^2+t_3^2))}{(u_j^2-u_3^2+t_3^2)^2} + \dfrac{|t_3||u_i^2(u_j^2-u_3^2+t_3^2)+u_j^2-u_3^2-t_3^2|}{(u_j^2-u_3^2+t_3^2)^2}\sqrt{\dfrac{u_3^2-u_j^2+u_j^2(u_j^2-u_3^2+t_3^2)}{(u_j^2-u_3^2)(u_i^2(u_j^2-u_3^2+t_3^2)-t_3^2)}} < 1 \\ \dfrac{(-1+u_i^2)(2u_j^2-u_j^4-2u_3^2+u_3^4-2(1+u_3^2)t_3^2+t_3^4+(u_j^2-u_3^2+t_3^2)^2(2\cos^2\alpha_2-1))}{2(1+u_i^2)(u_j^2-u_3^2)-2(1-u_i^2)t_3^2} \geqslant 0 \end{cases}$$

or

$$\begin{cases} \cos^2\alpha_2 = \dfrac{(u_3^2-u_j^2+t_3^2+u_j^2(u_j^2-u_3^2+t_3^2))}{(u_j^2-u_3^2+t_3^2)^2} - \dfrac{|t_3||u_i^2(u_j^2-u_3^2+t_3^2)+u_j^2-u_3^2-t_3^2|}{(u_j^2-u_3^2+t_3^2)^2}\sqrt{\dfrac{u_3^2-u_j^2+u_j^2(u_j^2-u_3^2+t_3^2)}{(u_j^2-u_3^2)(u_i^2(u_j^2-u_3^2+t_3^2)-t_3^2)}} > 0 \\ \dfrac{(-1+u_i^2)(2u_j^2-u_j^4-2u_3^2+u_3^4-2(1+u_3^2)t_3^2+t_3^4+(u_j^2-u_3^2+t_3^2)^2(2\cos^2\alpha_2-1))}{2(1+u_i^2)(u_j^2-u_3^2)-2(1-u_i^2)t_3^2} \geqslant 0. \end{cases}$$

i.e., $\Omega = 0$ has the solutions $\alpha_2 = \alpha_2^0 \neq \frac{m\pi}{2}$ ($m = 1,2$) for some i and j, but we require that $\alpha_1 = \frac{\pi}{2}$, $\beta_1 = \frac{(i-1)\pi}{2}$, $\beta_2 = \frac{(j-1)\pi}{2}$, $\alpha_2 = \alpha_2^0$ are not the maximum points of S_1.

For condition (II): when $\alpha_1 = 0$, $\beta_2 = \frac{(j-1)\pi}{2}$ ($j = 1,2$), $\Omega = 0$ is actually the equation satisfied by $\cos\alpha_2$.

Let $r_1 = \sqrt{((1+t_3)^2-u_3^2)((1-t_3)^2-u_3^2)}$, $r_2 = u_3^2+t_3^2+(u_3^2-t_3^2)^2+u_j^2(-1-u_3^2+t_3^2)$, we can prove that the following conditions are equivalent to (II).

2a1)

$$\begin{cases} u_j^2 < u_3^2 \quad \text{or} \quad (u_3^2+u_j^2(u_j^2-1-u_3^2+t_3^2))(r_2^2-r_1^2(u_j^2-u_3^2+t_3^2)) < 0,\ j=1 \\ u_j^2 < u_3^2 \quad \text{or} \quad (u_3^2+u_j^2(u_j^2-1-u_3^2+t_3^2))(r_2^2-r_1^2(u_j^2-u_3^2+t_3^2)) < 0,\ j=2 \end{cases}$$

If the conditions in 2a1) are not satisfied, i.e.

2a2) $u_j^2 \geqslant u_3^2$ and $(u_3^2+u_j^2(u_j^2-1-u_3^2+t_3^2))(r_2^2-r_1^2(u_j^2-u_3^2+t_3^2)) \geqslant 0$, $j = 1$ or $j = 2$ or $j = 1,2$, but

$$\begin{cases} \dfrac{u_3^2+t_3^2+u_j^2(u_j^2-1-u_3^2+t_3^2)}{(u_j^2-u_3^2+t_3^2)^2} + \dfrac{2|r_2t_3|}{(u_j^2-u_3^2+t_3^2)^2}\sqrt{\dfrac{u_3^2+u_j^2(u_j^2-1-u_3^2+t_3^2)}{r_2^2-r_1^2(u_j^2-u_3^2+t_3^2)}} > 1 \\ \dfrac{u_3^2+t_3^2+u_j^2(u_j^2-1-u_3^2+t_3^2)}{(u_j^2-u_3^2+t_3^2)^2} + \dfrac{2|r_2t_3|}{(u_j^2-u_3^2+t_3^2)^2}\sqrt{\dfrac{u_3^2+u_j^2(u_j^2-1-u_3^2+t_3^2)}{r_2^2-r_1^2(u_j^2-u_3^2+t_3^2)}} < 0 \end{cases}$$

If the conditions in 2a1) and 2a2) are not satisfied, i.e.
2a3) $u_j^2 \geqslant u_3^2$ and $(u_3^2+u_j^2(u_j^2-1-u_3^2+t_3^2))(r_2^2-r_1^2(u_j^2-u_3^2+t_3^2)) \geqslant 0$, $j=1$ or $j=2$ or $j=1,2$, but

$$\begin{cases} \cos^2\alpha_2 = \dfrac{u_3^2+t_3^2+u_j^2(u_j^2-1-u_3^2+t_3^2)}{(u_j^2-u_3^2+t_3^2)^2} + \dfrac{2|r_2t_3|}{(u_j^2-u_3^2+t_3^2)^2}\sqrt{\dfrac{u_3^2+u_j^2(u_j^2-1-u_3^2+t_3^2)}{r_2^2-r_1^2(u_j^2-u_3^2+t_3^2)}} \leqslant 1 \\ \dfrac{(1-u_j^2)(u_j^2-u_3^2)-(1+u_j^2)t_3^2+(u_j^2-u_3^2+t_3^2)^2\cos\alpha_2^2}{u_3^2+t_3^2+(u_3^2-t_3^2)^2+u_j^2(-1-u_3^2+t_3^2)} < 0 \end{cases}$$

or

$$\begin{cases} \cos^2\alpha_2 = \dfrac{u_3^2+t_3^2+u_j^2(u_j^2-1-u_3^2+t_3^2)}{(u_j^2-u_3^2+t_3^2)^2} - \dfrac{2|r_2t_3|}{(u_j^2-u_3^2+t_3^2)^2}\sqrt{\dfrac{u_3^2+u_j^2(u_j^2-1-u_3^2+t_3^2)}{r_2^2-r_1^2(u_j^2-u_3^2+t_3^2)}} \geqslant 0 \\ \dfrac{(1-u_j^2)(u_j^2-u_3^2)-(1+u_j^2)t_3^2+(u_j^2-u_3^2+t_3^2)^2\cos\alpha_2^2}{u_3^2+t_3^2+(u_3^2-t_3^2)^2+u_j^2(-1-u_3^2+t_3^2)} < 0 \end{cases}$$

If ρ_X satisfies conditions in 2a1) or 2a2) or 2a3), we find $\Omega = 0$ does not have solutions for $\alpha_1 = 0$, $\beta_2 = \frac{(j-1)\pi}{2}$.
If both 2a1), 2a2) and 2a3) are not satisfied, i.e.
2b) $u_j^2 \geqslant u_3^2$ and $(u_3^2+u_j^2(u_j^2-1-u_3^2+t_3^2))(r_2^2-r_1^2(u_j^2-u_3^2+t_3^2)) \geqslant 0$, $j=1$ or $j=2$ or $j=1,2$, but

$$\begin{cases} \cos^2\alpha_2 = \dfrac{u_3^2+t_3^2+u_j^2(u_j^2-1-u_3^2+t_3^2)}{(u_j^2-u_3^2+t_3^2)^2} + \dfrac{2|r_2t_3|}{(u_j^2-u_3^2+t_3^2)^2}\sqrt{\dfrac{u_3^2+u_j^2(u_j^2-1-u_3^2+t_3^2)}{r_2^2-r_1^2(u_j^2-u_3^2+t_3^2)}} = 1 \\ \dfrac{(1-u_j^2)(u_j^2-u_3^2)-(1+u_j^2)t_3^2+(u_j^2-u_3^2+t_3^2)^2}{u_3^2+t_3^2+(u_3^2-t_3^2)^2+u_j^2(-1-u_3^2+t_3^2)} \geqslant 0 \end{cases}$$

or

$$\begin{cases} \cos^2\alpha_2 = \dfrac{u_3^2+t_3^2+u_j^2(u_j^2-1-u_3^2+t_3^2)}{(u_j^2-u_3^2+t_3^2)^2} - \dfrac{2|r_2t_3|}{(u_j^2-u_3^2+t_3^2)^2}\sqrt{\dfrac{u_3^2+u_j^2(u_j^2-1-u_3^2+t_3^2)}{r_2^2-r_1^2(u_j^2-u_3^2+t_3^2)}} = 0 \\ \dfrac{(1-u_j^2)(u_j^2-u_3^2)-(1+u_j^2)t_3^2}{u_3^2+t_3^2+(u_3^2-t_3^2)^2+u_j^2(-1-u_3^2+t_3^2)} \geqslant 0 \end{cases}$$

i.e., $\Omega = 0$ only has the solution $\alpha_2 = \frac{m\pi}{2}$ $(m=0,1)$ for $\alpha_1 = 0$, $\beta_2 = \frac{(j-1)\pi}{2}$.
2c) $u_j^2 \geqslant u_3^2$ and $(u_3^2+u_j^2(u_j^2-1-u_3^2+t_3^2))(r_2^2-r_1^2(u_j^2-u_3^2+t_3^2)) \geqslant 0$, $j=1$ or $j=2$ or $j=1,2$, but

$$\begin{cases} \cos^2\alpha_2 = \dfrac{u_3^2+t_3^2+u_j^2(u_j^2-1-u_3^2+t_3^2)}{(u_j^2-u_3^2+t_3^2)^2} + \dfrac{2|r_2t_3|}{(u_j^2-u_3^2+t_3^2)^2}\sqrt{\dfrac{u_3^2+u_j^2(u_j^2-1-u_3^2+t_3^2)}{r_2^2-r_1^2(u_j^2-u_3^2+t_3^2)}} < 1 \\ \dfrac{(1-u_j^2)(u_j^2-u_3^2)-(1+u_j^2)t_3^2+(u_j^2-u_3^2+t_3^2)^2\cos\alpha_2^2}{u_3^2+t_3^2+(u_3^2-t_3^2)^2+u_j^2(-1-u_3^2+t_3^2)} \geqslant 0 \end{cases}$$

or

$$\begin{cases} \cos^2\alpha_2 = \dfrac{u_3^2+t_3^2+u_j^2(u_j^2-1-u_3^2+t_3^2)}{(u_j^2-u_3^2+t_3^2)^2} - \dfrac{2|r_2t_3|}{(u_j^2-u_3^2+t_3^2)^2}\sqrt{\dfrac{u_3^2+u_j^2(u_j^2-1-u_3^2+t_3^2)}{r_2^2-r_1^2(u_j^2-u_3^2+t_3^2)}} > 0 \\ \dfrac{(1-u_j^2)(u_j^2-u_3^2)-(1+u_j^2)t_3^2+(u_j^2-u_3^2+t_3^2)^2\cos\alpha_2^2}{u_3^2+t_3^2+(u_3^2-t_3^2)^2+u_j^2(-1-u_3^2+t_3^2)} \geqslant 0 \end{cases}$$

i.e., $\Omega = 0$ has the solution $\alpha_2 = \alpha_2^1 \neq \frac{m\pi}{2}$ for $\alpha_1 = 0$, $\beta_2 = \frac{(j-1)\pi}{2}$, but we require that $\alpha_1 = 0$, $\beta_2 = \frac{(j-1)\pi}{2}$ and $\alpha_2 = \alpha_2^1$ are not the maximum of S_1.

For condition (III): If $t_3^4 \neq (u_1^2-u_3^2)(u_2^2-u_3^2)$, when $\beta_1 = \frac{k\pi}{2}$, $\beta_2 = \frac{k\pi}{2}$, Δ and Ω can not be 0 simultaneously, then (4) does not have solutions. □

0.3 Proof of Corollaries

Proof of Corollary 1: For the states ρ_{zero}, the positivity of density matrix gives the conditions $(a_3-b_3)^2+(c_1+c_2)^2 \leqslant (1-c_3)^2$ and $(a_3+b_3)^2+(c_1-c_2)^2 \leqslant (1+c_3)^2$.

Case I: The maximal value of $\Delta_1 = u_1^2+u_2^2-1$

From the condition $(a_3-b_3)^2+(c_1+c_2)^2 \leqslant (1-c_3)^2$ and $(a_3+b_3)^2+(c_1-c_2)^2 \leqslant (1+c_3)^2$, we find that $c_1^2+c_2^2 \leqslant 1+c_3^2-a_3^2-b_3^2$. Hence, $u_1^2+u_2^2-1 \leqslant \frac{c_3^2-a_3^2}{1-b_3^2}$. $\frac{c_3^2-a_3^2}{1-b_3^2}$ gets the maximum value for small a_3^2 and large b_3^2. Due to $(a_3-b_3)^2+(c_1+c_2)^2 \leqslant (1-c_3)^2$ and $(a_3+b_3)^2+(c_1-c_2)^2 \leqslant (1+c_3)^2$, we obtain $|a_3-b_3| \leqslant 1-c_3$ and $|a_3+b_3| \leqslant 1+c_3$. When $a_3=0$, b_3 attains its maximum value, $\min\{1-c_3, 1+c_3\}$. Therefore,

$$\frac{c_3^2-a_3^2}{1-b_3^2} \leqslant \frac{c_3^2}{1-\min\{(1-c_3)^2,(1+c_3)^2\}} \leqslant |c_3|.$$

Actually when $c_3 \geqslant 0$, we find $u_1^2+u_2^2-1 \to c_3$ if $b_3 \to -1$ and $a_3-b_3 = 1-c_3$. When $c_3<0$, we find $u_1^2+u_2^2-1 \to |c_3|$ if $b_3 \to 1$ and $a_3+b_3 = 1+c_3$.

Case II: The maximal value of $\Delta_2 = \frac{1}{2}[u_1^2(u_3^2-t_3^2)+u_1^2+u_3^2+t_3^2-1-(1-u_1^2)\sqrt{((1-t_3)^2-u_3^2)((1+t_3)^2-u_3^2)}]$

For any given a_3, b_3 and c_3, Δ_2 increases with $|c_1|$. The maximum value of c_1 is attained when $(a_3-b_3)^2+(c_1+c_2)^2 = (1-c_3)^2$ and $(a_3+b_3)^2+(c_1-c_2)^2 = (1+c_3)^2$. We only need to consider the parameters a_3, b_3, c_1, c_2 and c_3 which satisfy $(a_3-b_3)^2+(c_1+c_2)^2 = (1-c_3)^2$ and $(a_3+b_3)^2+(c_1-c_2)^2 = (1+c_3)^2$. Let $\Gamma_1 = \sqrt{(1-c_3)^2-(a_3-b_3)^2}$, and $\Gamma_2 = \sqrt{(1+c_3)^2-(a_3+b_3)^2}$. We assume $c_1 \geqslant c_2$, and $c_1 \geqslant 0$, then $c_2 \leqslant \frac{\Gamma_1}{2}$. Set $c_2 = \frac{\Gamma_1}{2}-x$, $c_1 = \frac{\Gamma_1}{2}+x$, for $0 \leqslant x \leqslant \frac{\Gamma_2}{2}$. We have that Δ_2 is an increasing function of x. Hence,

$$\Delta_2 \leqslant \frac{c_3^2-a_3^2}{2(1-b_3^2)} \frac{1-b_3^2+c_3^2-a_3^2+\Gamma_1\Gamma_2}{1-b_3^2}.$$

$\Gamma_1\Gamma_2/(1-b_3^2)$ attain the maximum value when $c_3 \geqslant 0$ $(\leqslant 0)$ and $a_3b_3 \geqslant 0$ $(\leqslant 0)$. By the optimization method, one can find $\frac{c_3^2-a_3^2}{2(1-b_3^2)} \frac{1-b_3^2+c_3^2-a_3^2+\Gamma_1\Gamma_2}{1-b_3^2}$ attain the maximum value when $1+c_3 = |a_3+b_3|$ or $1-c_3 = |a_3-b_3|$. So we have when b_3 approaches to -1 and $a_3-b_3 = 1-c_3$, or $b_3 \to 1$ and $a_3+b_3 = 1+c_3$, we have the maxima of $\Delta_2 = |c_3|$.

We have the steerability $S \leqslant |c_3|$ when $b_3 \to -1$ and $a_3-b_3 = 1-c_3$, or $b_3 \to 1$ and $a_3+b_3 = 1+c_3$. Then either $c_1 = -c_2 = \pm\sqrt{(1+b_3)(c_3-b_3)}$ or $c_1 = c_2 = \pm\sqrt{(1-b_3)(b_3-c_3)}$, and $S \leqslant \frac{N}{2}$. This completes the proof. □

Proof of Corollary 2: Due to the positivity of density matrix ρ_X, a_3, b_3 and c_i $(i=1,2,3)$ satisfy the conditions $|a_3+b_3| \leqslant \sqrt{(1+c_3)^2-(c_1-c_2)^2}$ and $|a_3-b_3| \leqslant \sqrt{(1-c_3)^2-(c_1+c_2)^2}$. Let

$$\begin{aligned}\Omega =&\{|a_3+b_3| \leqslant \sqrt{(1+c_3)^2-(c_1-c_2)^2},\\ &|a_3-b_3| \leqslant \sqrt{(1-c_3)^2-(c_1+c_2)^2}\}\end{aligned}$$

The minimum of $\max\limits_{\alpha_i,\beta_i} S_1$ is attained at the interior points or the boundary of Ω for given CHSH value N:

(1) For the interior points of Ω, we find the minimal steerability $S = \frac{N^2}{4}-1$ when $t_3 = 0$, $u_3 = c_3$, and $b_3 = 0$.

(2) For the boundary of Ω, the minimal value of S is attained at the extreme points of Δ_2 or Δ_3, or the points solving the equation $\Delta_2 = \Delta_1$, $\Delta_3 = \Delta_1$, or $\Delta_2 = \Delta_3$. By numerical simulations, we find that when $N \geqslant 2$, the lower bound is very close to $\frac{N^2}{4}-1$ but smaller than $\frac{N^2}{4}-1$. □

0.4 Matlab program for computing steerability of general two-qubit states

opts=optimoptions(@fmincon,'Algorithm','interior
−point');
problem=createOptimProblem(opt);
gs=GlobalSearch;
[x,f]=run(gs,problem).
Here
opt='fmincon','objective',... $@(x)(-S(x_1,x_2,x_3,x_4))$, '$x_0'$, x^*, 'lb', lb^*, 'ub', un^*, 'options',opts
with $x_1 = \alpha_1, x_2 = \beta_1, x_3 = \alpha_2, x_4 = \beta_2$, $x^* = [0,0,0,0]$, $lb^* = [0,0,0,0]$, and $ub^* = [\pi, 2\pi, \pi, 2\pi]$.
